# Identifying individuals at risk of needing CKD associated medications in a European kidney disease cohort

**DOI:** 10.1186/s12882-024-03497-y

**Published:** 2024-02-20

**Authors:** Eleni Stamellou, Turgay Saritas, Marc Froissart, Florian Kronenberg, Peter Stenvinkel, David C. Wheeler, Kai-Uwe Eckardt, Jürgen Floege, James Fotheringham

**Affiliations:** 1 Division of Nephrology and Clinical Immunology, RWTH University of Aachen, Aachen, Germany; 2 Department of Nephrology, University Hospital of Ioannina, Ioannina, Greece; 3 Centre de Recherche Clinique (CRC), Lausanne University Hospital, Lausanne, Switzerland; 4 Department of Genetics, Institute of Genetic Epidemiology, Medical University of Innsbruck, Innsbruck, Austria; 5 Division of Renal Medicine, Department of Clinical Science, Intervention and Technology, Karolinska Institutet, Stockholm, Sweden; 6 Department of Renal Medicine, University College London, London, United Kingdom; 7 Department of Nephrology and Medical Intensive Care, Charité-Universitätsmedizin Berlin, Berlin, Germany; 8 Northern General Hospital, Sheffield Kidney Institute, Herries Road, Sheffield, South Yorkshire S5 7AU United Kingdom; 9 School of Health and Related Research, University of Sheffield, Sheffield, United Kingdom

**Keywords:** CKD G4/G5, CKD-MBD, Renal anemia, ESAs, VDRA, Phosphate binders

## Abstract

**Background:**

The consequences of chronic kidney disease (CKD) can be addressed with a range of pharmacotherapies primarily prescribed by nephrologists. More accurate information regarding future CKD-related pharmacotherapy requirements could guide clinical decisions including follow-up frequency.

**Methods:**

Following assignment to derivation and validation groups (2,1), variables predicting individually future use of vitamin D receptor agonists (VDRA), phosphate binders, erythropoiesis stimulating agents (ESAs) and iron were identified using logistic regression in a prospective cohort study containing demography, comorbidity, hospitalization, laboratory, and mortality data in patients with CKD stage G4/G5 across six European countries. Discriminative ability was measured using C-statistics, and predicted probability of medication use used to inform follow-up frequency.

**Results:**

A total of 2196 patients were included in the analysis. During a median follow-up of 735 days 648 initiated hemodialysis and 1548 did not. Combinations of age, diabetes status and iPTH, calcium, hemoglobin and serum albumin levels predicted the use of ESA, iron, phosphate binder or VDRA, with C-statistics of 0.70, 0.64, 0.73 and 0.63 in derivation cohorts respectively. Model performance in validation cohorts were similar. Sixteen percent of patients were predicted to have a likelihood of receiving any of these medications of less than 20%.

**Conclusions:**

In a multi-country CKD cohort, prediction of ESA and phosphate binder use over a two-year period can be made based on patient characteristics with the potential to reduce frequency of follow-up in individuals with low risk for requiring these medications.

**Supplementary Information:**

The online version contains supplementary material available at 10.1186/s12882-024-03497-y.

## Background

Chronic kidney disease (CKD) is a major public health problem associated with poor quality of life, and high morbidity and mortality rate [1]. Despite improvements in dialysis care, the mortality of patients treated by dialysis remains unacceptably high. Suboptimal care during CKD stages G4/CKD G5ND may contribute to this, as it has been shown that longer duration of specialized nephrology care prior to transition to chronic dialysis, is associated with significantly better outcomes [2, 3]. CKD care includes management for anemia and disturbances of bone mineral metabolism (CKD associated bone mineral disease; CKD-MBD). These conditions are common in patients with advanced CKD and are associated with adverse clinical outcomes, including cardiovascular events, protein energy wasting and death [4–9]. As anemia and CKD-MBD represent modifiable risk factors for cardiovascular and renal disease progression, early recognition and treatment represent a key task for nephrologists [10–14].

Recognizing individuals requiring CKD-related pharmacotherapy in the future helps the nephrologist identify a more severe CKD-phenotype requiring more intensive monitoring, management, or support than CKD severity stratification using eGFR alone. However, no predictive models exist to guide the need or intensity for CKD-related pharmacotherapy. Physicians must therefore make uninformed or inconsistent decisions about which patients to monitor more closely, risking delays in treatment in those who ultimately need a more intense nephrological care, or conducting unnecessary nephrological visits in those who do not need them. More targeted nephrological visits and treatments might reduce the burden of disease and save nephrology resources.

In this study we identified baseline patient characteristics and laboratory values to predict the need for traditionally nephrologist-led pharmacotherapy of renal anemia and CKD-MBD during follow-up, to inform predictive models which could support clinical decision-making. These models identified more severe CKD phenotypes, and conversely enable reduced follow-up frequency in milder CKD phenotypes.

## Methods

### Study population

The Analyzing Data, recognizing Excellence and Optimizing Outcomes (ARO) cohort III study contains anonymized longitudinal individual-level data for pre-hemodialysis patients (N = 2471) who received pre-dialysis care in Fresenius Medical Care (FMC) facilities across six European countries (Italy; Czech Republic; Serbia; Bosnia; Slovak Republic and Russia) between 2012 and 2014 and who were followed until the end of 2016. Informed consent was obtained from all patients by FMC. Data on demography, comorbidity, laboratory, and outcomes such as dialysis, death and transplantation were captured prospectively in the FMC database. All local ethical and regulatory obligations concerning patient data for each of the 6 participating countries were met. The study has been approved by the institutional review board of the Medical University of Innsbruck (EK-Nr. 1339/2020). Follow-up commenced on the date of patients’ first referral to an FMC unit until December 31, 2016. Chronic dialysis was defined as receiving hemodialysis for more than one month.

### Follow-up, endpoints and adjustment variables

In the present analysis, patients with CKD stage 4/5 (eGFR < 30 ml/min/1.73m^2^), being managed in healthcare systems where their first observation in the dataset represented their first assessment by a nephrologistwere included and were followed-up until they transitioned to chronic hemodialysis or until the end of follow-up (December 2016). Endpoints of interest were medication requirements when patients transitioned to chronic hemodialysis or at the end of follow-up: erythropoiesis stimulating agents (ESAs), iron (both oral and i.v), vitamin D receptor agonists (VDRA) and phosphate binders. Variables included demographic variables, including age (< 49; 50–60; 61–70; 71–80 and > 80 years old), sex (male; female), body mass index (BMI; underweight: < 18.5; normal range: 18.5–25, overweight: 25.01–30; obesity > 30 kg/m^2^), smoking (former and current smokers; nonsmokers), country (Italy; Czech Republic; Serbia; Bosnia; Slovak Republic and Russia); and comorbid conditions (diabetes, clinical diagnosis of hypertension, cardiovascular disease, cancer and etiology of kidney disease). Comorbid conditions and medications were categorized as present or absent at the time of the first visit in an FMC unit. Serum laboratory variables included: hemoglobin (categorized as < 100; 100–120; > 120 g/l), serum phosphate (< 0.8; 0.8–1.49; ≥ 1.5 mmol/l), total calcium (< 2.1; 2.1–2.6; > 2.6 mmol/l), intact parathormone (iPTH) (< 149; 150–300; > 300 ng/l) and serum albumin (≤ 35 g/l; > 35). Medications recorded at the initial nephrology visit or when patients transition to hemodialysis or at the end of follow-up were ESAs, iron, VDRA, phosphate binders, diuretics, RAASi and antihypertensives. Glomerular filtration rate was estimated (eGFR) using the Chronic Kidney Disease Epidemiology (CKD-EPI) 2009 creatinine-based equation.

Through consensus among seven independent nephrologists and considering the absence of prior literature on this matter, participants with a than 20% for requiring these medications over the next 20 months risk should be classified as low-risk individuals.

### Statistical analysis

Baseline patient characteristics were reported using descriptive statistics. Continuous variables were described using means and standard deviations or median and interquartile range; categorical data were reported as counts and frequencies. Intergroup comparisons were performed using the Pearson *chi-square test, Student’s t-test.* Derivation and validation cohorts were assigned randomly with a 2:1 ratio. After excluding all patients receiving the relevant CKD-related pharmacotherapy (ESA, VDRA, phosphate binders), binary logistic regression analyses were employed on the derivation cohort to estimate the associations between baseline characteristics, and individually the future prescription of ESAs, iron (both oral and i.v), VDRA and phosphate binders. While cause-specific Cox models are commonly employed in time to event analyses, they are prone to overestimating risk when censoring for a competing risk (e.g. death) [15], and is recognized as a weakness of some widely adopted risk [16]. Patients who were already under treatment were excluded from the corresponding analysis. The reference values of covariates in our regression model were age: 71–80 years old, male gender, BMI 18.5–25 kg/m^2^, non-smokers, diabetes mellitus as primary renal disease, hemoglobin 100–120 g/l, serum total albumin > 35 g/l, serum-calcium 2.1–2.6 mmol/l, serum-phosphate 0.8–1.5 mmol/l, iPTH < 150 ng/l. Backward selection was employed to retain predictive variables (*p* < 0.05) prior to the prediction of individual probabilities of each of the four CKD pharmacotherapies. For the evaluation of our predictive model’s discrimination ability, we calculated C-statistics. We inputted the predictor variables into the model and obtained the predicted probabilities for each patient. Values over 0.7 were considered indicative for a good model. Finally, we ran the model to predict any requirement on the above-mentioned medications.

### The formulae to calculate the predicted probability of starting treatment

$${\text{F}}\left({\text{x}}\right)={{\text{e}}}^{{\text{X}}}/{{\text{e}}}^{{\text{x}}}+1$$

Where X is estimated by summing the coefficients associated with the presence or absence of the predictor variables (full equations provided in supplemental materials).

Sensitivity analyses were performed excluding CKD5 patients, but resulted in inferior predictive performance and are not reported. Statistical analysis was performed using SPSS 28.0 and R version 4.1.0. A *p* < 0.05 was considered statistically significant.

## Results

### Study population

**Fig. 1:**
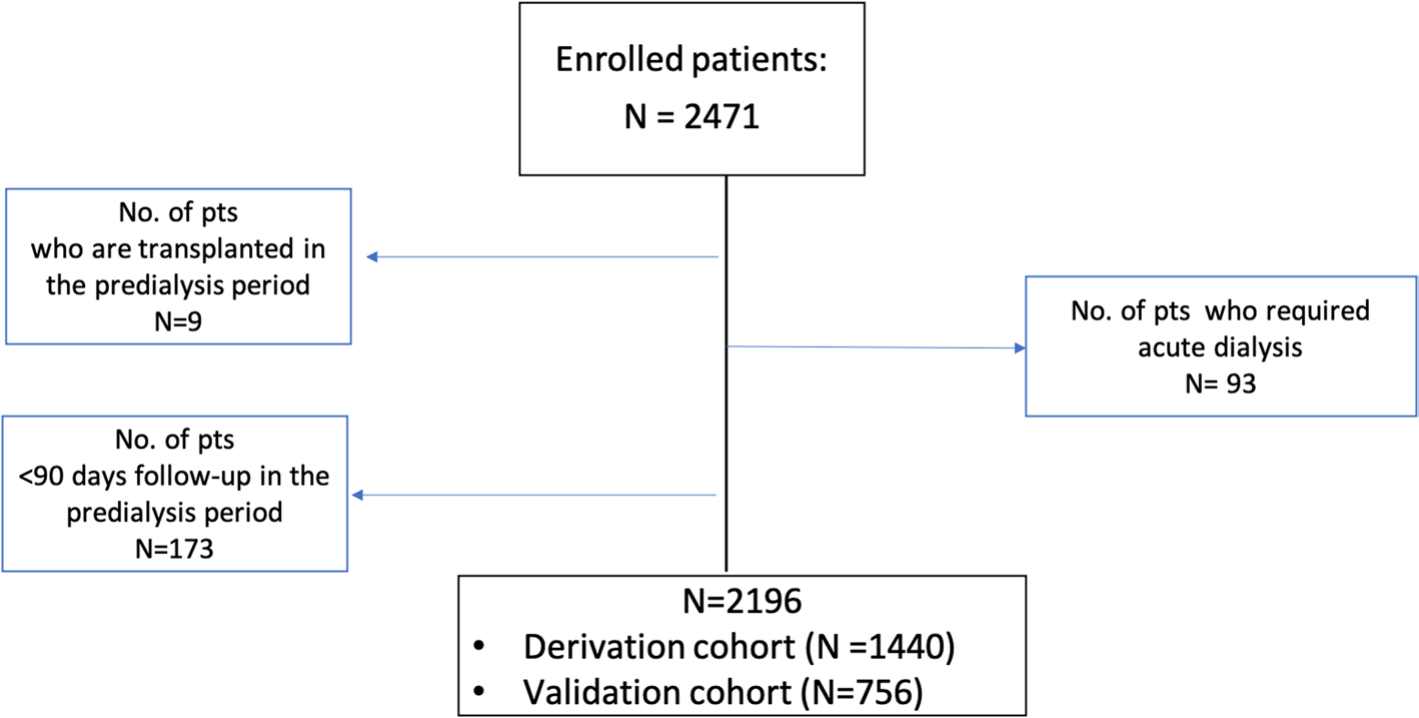
Study flow chart

Between April 1, 2012 and June 30, 2014, 2471 patients with eGFR < 30 ml/min/1.73m^2^ were recruited. A total of 2196 patients were included in the present analysis. Patients who were transplanted during the pre-dialysis period (n = 9), with < 90 days of follow-up in the pre-dialysis period (n = 173), and who were received temporary dialysis (n = 93) were excluded, as medication requirements were unobserved or changed during follow-up. Of these, 648 patients (29.5%) transitioned to chronic hemodialysis and 1548 did not, while 334 died during a median of 735 days follow-up period (Fig. 1). Among enrolled patients, the mean age was 69 years, and 52% were women. Almost half of the patients had a history of hypertension and one third history of diabetes. Diabetic nephropathy followed by hypertensive nephropathy were the most common causes of CKD. At baseline, eGFR was 18.6 ml/min/1.73m^2^. Half of the patients were on a diuretic (51.7%) at referral and 33.7% were on RAASi. The characteristics of the derivation and validation groups are presented in Table 1. The two groups exhibited no significant differences and shared similar traits with the entire cohort. Patient already under treatment with CKD medications at referral displayed lower hemoglobin and eGFR, along with higher phosphate and iPTH (suppl. Table 1).

**Table 1:** Baseline characteristics

	**Whole cohort** **(N = 2196)**	**Derivation cohort** **(N = 1440)**	**Validation cohort** **(N = 756)**
*Age at baseline (years)*	69 ± 13	68.8 ± 13.2	69.2 ± 13.2
*Gender*			
Female	1138 (51.8)	745 (51.7)	393 (52.0)
Male	1058 (48.2)	695 (48.3)	363 (48.0)
*Body mass index (kg/m*^*2*^*)*	29 ± 5.9	29 ± 5.8	29.1 ± 6.0
*missing*	*70 (3.2)*	*44 (3.0)*	*26 (3.4)*
*Smoking status*			
Nonsmoker	1140 (51.9)	761 (52.8)	379 (50.1)
Former	430 (19.6)	275 (19.1)	155 (20.5)
Current	215 (9.8)	141 (9.8)	74 (9.8)
Missing	411 (18.7)	263 (18.3)	148 (19.6)
*History of cancer*	106 (4.8)	70 (4.9)	36 (4.8)
*History of CVD*	492 (22.4)	338 (23.5)	154(20.4)
*History of diabetes*	*772 (35.2)*	*517 (35.9)*	255 (33.7)
*History of hypertension*	*1022 (46.5)*	*686 (47.6)*	336 (44.4)
*Chronic kidney disease etiology*			
Hypertensive nephropathy	46 (21.7)	331 (23.0)	145 (19.2)
Glomerulonephritis	146 (6.6)	91 (6.3)	55 (7.3)
Diabetic nephropathy	530 (24.1)	348 (24.2)	182 (24.1)
Tubulo-interstitial	347 (15.8)	224 (15.6)	123 (16.3)
Polycystic kidney disease	84 (3.8)	50 (3.5)	34 (4.5)
Miscellaneous/other	477 (21.7)	313 (21.7)	164 (21.7)
Missing	136 (6.2)	83 (5.8)	53 (7.0)
*Country*			
Italy	526 (24)	334 (23.2)	192 (25.4)
Czech Republic	706 (32.1)	465 (32.3)	241 (31.9)
Serbia	123 (5.6)	85 (5.9)	38 (5.0)
Bosnia	56 (2.5)	35 (2.4)	21 (2.8)
Slovak Republic	625 (28.5)	412 (28.6)	*213 (28.2)*
Russia	160 (7.3)	109 (7.6)	51 (6.7)
*Iron at referral*	385 (17.5)	251 (17.4)	134 (17.7)
*ESA at referral*	285 (13.0)	189 (13.1)	96 (12.7)
*VDRA therapy at referral*	611 (27.8)	401 (27.8)	210 (27.8)
*Phosphate binders at referral*	287 (13.1)	189 (13.1)	98 (13.0)
*Amount of antihypertensives at referral*			
*0*	989 (45.0)	646 (44.9)	343 (45.4)
*1–2*	1086 (49.5)	707 (49.1)	379 (50.1)
*More than 3*	121 (5.4)	87 (6.1)	34 (4.5)
*RAASi at referral*	739 (33.7)	484 (33.6)	255 (33.7)
*Diuretic at referral*	1133 (51.6)	756 (52.5)	377 (49.9)
*Hemoglobin(g/l)*	116 ± 16	116.2 ± 16	116.9 ± 16.8
Missing	*186 (8.5)*	*116 (8.0)*	*70 (9.3)*
*Ferritin (μg/l)*	276 (139, 524)	271 (142, 504)	280 (136,564)
Missing	822 (37.4)	544 (37.8)	*278 (36.8)*
Transferrin saturation (TSAT)	20.3 (15, 26)	20.0 (15, 26)	21(15, 27)
Missing	*1315 (60.0)*	*860 (60.0)*	*455 (60.2)*
*Serum albumin (g/l)*	40.6 ± 4.4	40.5 ± 4.5	40.6 ± 4.4
Missing	*482 (21.9)*	*308 (21.4)*	*174 (23.0)*
*Total calcium (mmol/l)*	2.3 ± 0.18	2.3 ± 0.18	2.3 ± 0.18
Missing	*273 (12.4)*	*184 (12.8)*	*89 (11.8)*
*Phosphate (mmol/l)*	1.3 ± 0.29	1.3 ± 0.28	1.3 ± 0.3
Missing	*285 (13.0)*	*189 (13.0)*	*96 (12.7)*
*iPTH (ng/l)*	124 (72, 202)	125 (73, 201)	121 (72, 206)
Missing	*479 (21.8)*	*321 (22.3)*	158 (20.9)
*eGFR (CKD-EPI)*	18.6 ± 6.5	18.5 ± 6.5	18.8 ± 6.5
Missing	*243 (11.0)*	*156 (10.8)*	87 (11.5)
*Days of follow-up*	*735 (290, 1255)*	*733 (293, 1264)*	752 (283, 1237)

*CVD* Cardiovascular disease, *RAASi* Renin angiotensin aldosterone system inhibitors, *ESA* Erythropoesis Stimulating Agent, *VDRA* Vitamin D receptor agonists, *iPTH* intact parathormone, *HD* Hemodialysis

Data are expressed as mean ± SD or median, interquartile range as appropriate. Categorical variables are reported using n (%)

### The association between phenotype, laboratory variables and medication requirements

Following backward selection, independently predictive variables of CKD medications are shown in Table 2. Significant predictors for the prescription of ESAs included VDRA and iron therapy already at referral, hemoglobin levels < 120 g/l and iPTH levels > 150 ng/l (Table 2). Regarding iron therapy, predictors included low eGFR at baseline and treatment with ESAs already at referral. Regarding the route of iron administration, most patients were receiving oral iron at baseline (13.9% oral vs 3.4% IV), with IV iron use increasing at the end of follow-up (15.3% vs 5.8%). In patients transitioning to hemodialysis, intravenous iron therapy was more frequent compared to those staying off hemodialysis (17.4% vs 10.8%). The predictors for prescription of phosphate binders during the follow-up (Table 2) included younger age, serum albumin concentration > 35 g/l, baseline iPTH levels > 150 ng/l and hemoglobin < 120 g/l. Lastly, the prescription of VDRA therapy was associated with a history of diabetes, baseline iPTH > 150 ng/l, serum albumin > 35 g/l, and abnormal calcium levels (≥ 2.6 mmol/L) (Table 2).

**Table 2:** Multivariate logistic regression of the risk of requiring CKD-related pharmacotherapy during the pre-dialysis period

	**P value**	**OR**^**a**^** (95% CI)**
**Risk for requiring ESAs during the pre-dialysis period**		
VDRA at referral	0.052	1.61 (0.99–2.60)
Iron at referral	0.042	1.85 (1.02–3.35)
eGFR at referral	0.026	0.95 (0.92–0.99)
Hemoglobin ref. < 100 g/l	0.053	2.24 (0.99–4.60)
Hemoglobin ref. 100- 120 g/l	0.040	2.18(1.28–3.71)
iPTH ref. > 150 ng/l	0.044	1.66(1.01–2.73)
**Risk for requiring iron therapy during the pre-dialysis period**		
eGFR ref. (CKD-EPI)	< 0.001	0.93 (0.89–0.97)
ESAs at referral	0.058	2.02 (0.98–4.17)
**Risk for requiring phosphate binders during the pre-dialysis period**		
Age > 80	0.03	0.20 (0.07–0.58)
Age 50–60	0.09	0.47 (0.18–1.15)
Age 61–70	0.08	0.33 (0.15–0.75)
Age 71–80	0.02	0.28 (0.13–0.64)
iPTH ref. > 150 ng/l	0.03	2.33 (1.33–4.04)
Hemoglobin ref. < 100 g/l	0.009	2.75 (1.29–5.86)
Hemoglobin ref. 100- 120 g/l	0.70	1.13 (0.6–2.09)
Serum albumin > 35 g/l	0.10	2.46 (1.29–5.86)
eGFR ref. (CKD-EPI)	0.05	0.93 (0.90–0.98)
**Risk for requiring VDRA during the pre-dialysis period**		
History of diabetes	0.025	1.55 (1.06–4.77)
Serum albumin > 35 g/l	0.016	2.40 (1.18–4.91)
Calcium < 2.1 mmol/l	0.189	1.54 (0.81–2.93)
Calcium > 2.6 mmol/l	0.040	2.27 (1.04–4.96)
iPTH > 150 ng/l	< 0.001	3.2 (2.15–4.77)

*SESA* Erythropoesis Stimulating Agent, *CVD* Cardiovascular disease, *iPTH* intact parathormone, *VDRA* Vitamin D receptor agonists

^a^Odds rations (OR) are computed through binary logistic regression. Reference groups were age < 49, Calcium between 2.1 and 2.6 mmol/l, Parathormone below 150 ng/l, Albumin < 35 g/l, Hemoglobin > 120 g/l

### The prediction of future medication prescriptions

As depicted in Table 3, the discriminative performance varied across models. Regarding ESAs, both the derivation and validation cohorts exhibited good discrimination, with c-statistics of 0.70 and 0.73, respectively. Similar robustness was observed for phosphate binders, with c-statistics of 0.73 and 0.74, respectively. However, the c-statistics for both the validation and derivation cohorts displayed poor performance for iron and VDRA, yielding values of 0.64 (derivation), 0.63 (validation) and 0.66 (derivation), 0.69 (validation) respectively.

**Table 3:** C-statistic for the models predicting CKD-related pharmacotherapy

	**Area**	**95% CIs**
**ESAs**		
Derivation cohort	0.700	0.643–0.750
Validation cohort	0.728	0.652–0.803
**Iron**		
Derivation cohort	0.641	0.568–0.713
Validation cohort	0.630	0.545–0.715
**Phosphate binders**		
Derivation cohort	0.732	0.667–0.797
Validation cohort	0.741	0.663–0.819
**VDRA**		
Derivation cohort	0.659	0.619–0.716
Validation cohort	0.668	0.590–0.729

Model performance was assessed within specific patient groups: models for ESAs and iron exhibited a very good discrimination among patients with hemoglobin levels > 100 g/l at referral. Concerning phosphate binders, the c-statistic was 0.76 for patients with phosphate levels < 1.4 mmol/l at referral, and 0.59 for those with phosphate levels > 1.5 mmol/l (suppl. Table 2). Predictive factors for any CKD-related pharmacotherapy were also assessed, however, the model’s performance was poor, with a c-statistic of 0.65 and 0.67 respectively (suppl. Table 3 and 4). In our study cohort of CKD G4/5 predialysis patients, these models identified 353 out of 2196 individuals as having a risk of less than 20% risk for any of these medications. Similarly, among CKD G4 patients, we found that 291 out of 1314 exhibited a risk of less than 20%.

## Discussion

Our analysis provides a broad overview which patients to follow-up, aiming to reduce delays in treatment in those who ultimately need a more intense nephrological care. To our knowledge, this is the first study to describe the risk factors for initiation of CKD-related pharmacotherapy in pre-dialysis patients. Our aim is with the potential to inform clinical decisions around extending follow-up in individuals with low risk for requiring these medications.

The present study investigated risk factors for future medication use in a cohort of CKD4 patients, with a goal of predicting use of ESAs, iron (both oral and i.v), VDRA and phosphate binders. Age, history of diabetes, iPTH, hemoglobin, calcium and serum albumin levels predicted medication needs. The models showed varying prediction capabilities, which were best for ESAs and phosphate binders. A risk threshold of 20% was agreed to categorize low-risk patients needing these medications, identifying 353 such cases in the cohort.

Our findings shed light on several significant factors that influence the prescription of different medications, providing valuable insights for clinical practice and patient management. In our study, an association was observed between the baseline use of VDRA and increased iPTH levels and the prescription of ESAs. Indeed, several studies have found an association between CKD-MBD parameters and anemia [17, 18]. Together, these findings underscore the importance of monitoring and addressing CKD-MBD to reduce the need for ESAs [19–21].

The findings regarding phosphate binders were particularly intriguing. Older patients (> 60 years) were less likely to receive phosphate binders during follow-up. The relationship between age and serum phosphate levels in adults has been recognized for many years [22]. This age-related decline in serum phosphate levels has been attributed to changes in tubular phosphate reabsorption, which may, in turn, be explained by age-dependent alterations in tubular phosphate handling or in its hormonal regulators [23]. Additionally, another study observed a significant decrease in serum phosphorus levels with age in dialysis patients as well [24, 25]. One possible explanation is that relatively low caloric and protein intake is common among elderly HD patients. This observation highlights the role of nutritional status in phosphate management. As expected, malnourished patients were more susceptible to having low phosphorus levels [26]. However, this discrepancy might also reflect age-related differences in treatment priorities or tolerability, warranting further investigation.

Given that diabetic patients are known to develop anemia earlier, regardless of the stage of CKD [14, 27], it was unexpected we did not find a significant association between diabetes and the risk of prescribing ESAs. However, patients with a history of diabetes at enrollment were more likely to be treated with VDRA during the follow-up. In a cohort of pre-dialysis patients, diabetes mellitus was associated with CKD-MBD, including higher calcium-phosphorus product throughout all stages of CKD, poorer vitamin D status and lower serum calcitriol levels [28].

We acknowledge that future use of CKD-specific medications is one of a number of factors a healthcare professional is considering when evaluating the follow-up requirements of a person with kidney disease including the risk of kidney failure*.* Variation in regional guidelines and the quality of care among the different countries cannot be excluded. However, the observation that laboratory parameters associated with renal anemia and CKD-MBD did not vary by clinically relevant amounts should be reassuring (suppl. Table 5).

A strength of our analysis is that we used routinely available laboratory data in patients with CKD, mirroring what a nephrologist would have available to them in clinical practice. The variables in our analysis were carefully considered to avoid anything estimated from future variable observations, or that may require additional analysis by the healthcare professional, such as eGFR slope, that may be not feasible in all clinical settings. Our cohort is unique and encompassed a large population from various European countries and health-care systems. Limitations include that our study was based on data generated from a single commercial kidney care provider, and therefore it could be considered less generalizable to other chronic kidney disease populations. It is important to note that approximately 10% of the cohort selected for analysis had missing data precluding their inclusion in our multivariable models, but less than recently reporting prognostic kidney disease research [16]. Missingness also precluded the inclusion of albuminuria in our analyses.

Our study offers valuable insights for policy and clinical practice in managing CKD patients. Patients at a lower risk, could benefit from an extended follow-up schedule. This approach not only conserves healthcare resources but also allows healthcare professionals to allocate more time and intensive care to patients who require immediate attention. By tailoring the frequency of follow-up appointments based on risk levels, healthcare systems can optimize resource utilization and improve patient outcomes. While our analysis provides valuable insights into predicting medication requirements in patients with advanced CKD, it is crucial to acknowledge the need for trials to validate the effectiveness and potential inferiority of the suggested approach.

## Conclusions

Our study highlights the multifaceted nature of medication requirements in CKD patients. By identifying significant predictors for the initiation of specific pharmacotherapies, we provide a foundation for informed clinical decisions and policy development. With a holistic perspective, we aim to contribute to improved patient outcome and enhanced management of CKD-related complications.

### Supplementary Information

**Supplementary material 1.**

**Supplementary material 2.**

## Data Availability

The datasets generated and /or analysed during the current study are not publicly available due to the conditions stated at the time of patient consent, but are available from thee corresponding author upon reasonable request.

## Abbreviations

ARO: Recognizing Excellence and Optimizing Outcomes
CKD: Chronic kidney disease
VDRA: Vitamin D receptor agonists
ESAs: Erythropoiesis stimulating agents
CKD-MBD: CKD associated bone mineral disease
RAASi: Renin angiotensin aldosterone system inhibitors
BMI: Body mass index
CKD-EPI: Chronic Kidney Disease Epidemiology Collaboration
