# Supplementary material for: Identifying individuals at risk of needing CKD associated medications in a European kidney disease cohort
Source: BMC Nephrol. 2024 Feb 20;25:60. doi: 10.1186/s12882-024-03497-y (PMC10880231; doi:10.1186/s12882-024-03497-y)
Supplement: Supplementary file 1 — Supplementary material 1. [file 12882_2024_3497_MOESM1_ESM.docx]

*The formulae to calculate the predicted probability of starting treatment*

F(x) =e^X^/ e^x^+1

Where X is estimated by summing the coefficients associated with the presence or absence of the predictor variables (full equations provided in supplemental materials)

- ESA: x=(gender(male)*0.454) +(iron at referral *0.617)+ (eGFR*(-0.047)+ (Hb less than 100g/l* 0.759)+ (Hb (between 100-120g/l)*0.778)+(VDRAref *0.477)+(PTH>?? *0.509) -2.429
- Iron: (ESAref. *0.702)+(eGFR*(-0.078))-1.062
- Phosphate binders: (eGFR*(-0.068)) + (age >80*(-1.595)) + (age 61-70*(-1.097)) + (age 71-80*(-1.261) + (Hb less than 100g/l* 1.010) + (iPTH>150ng/l*0.84)-1.581
- VDRA: (History of Diabetes mellitus *0.437) *(Alb. >35g/l *0.877) + (iPTH>150ng/l*1.163) +(Ca>2.6mmol/l*0.820)-2.439
