# Supplementary material for: Identifying individuals at risk of needing CKD associated medications in a European kidney disease cohort
Source: BMC Nephrol. 2024 Feb 20;25:60. doi: 10.1186/s12882-024-03497-y (PMC10880231; doi:10.1186/s12882-024-03497-y)
Supplement: Supplementary file 2 — Supplementary material 2. [file 12882_2024_3497_MOESM2_ESM.docx]

**Suppl. Tables**

| **Suppl. Table 1. Baseline characteristics of patients being on treatment at referral versus not.** | | | | | | | | | | | | | | |
| --- | --- | --- | --- | --- | --- | --- | --- | --- | --- | --- | --- | --- | --- | --- |
|  | | **ESAs** | | | **Iron** | | | **Phosphate binders** | | | **VDRA** | | | |
|  | | No | Yes | p | No | Yes | p | No | Yes | p | No | No | Yes |  |
| S.-Albumin | 40.8±4.3 | | 39.4±5 | <0.001 | 40.7±4.4 | 40±4.4 | 0.036 | 40.6±4.4 | 40.3±4.4 | 0.2 | 40.6±4.5 | 40.4±4.4 | 0.226 |  |
| T.-Calcium | | 2.3±0.18 | 2.28±0.17 | <0.001 | 2.31±0.2 | 2.3±0.2 | <0.001 | 2.32±0.1 | 2.28±0.2 | 0.02 | 2.32±0.2 | 2.3±0.2 | 0.092 |  |
| Phosphate | | 1.3±0.3 | 1.4±0.3 | <0.001 | 1.3±0.3 | 1.4±0.3 | <0.001 | 1.28±0.26 | 1.47±0.3 | <0.001 | 1.29±0.3 | 1.35±0.3 | <0.001 |  |
| iPTH | | 161±164 | 200±217 | <0.001 | 157±156 | 210±230 | <0.001 | 159±162 | 214±220 | <0.001 | 151±143 | 194±220 | <0.001 |  |
| Hemoglobin | | 118±16 | 105±12 | <0.001 | 118±16 | 108±13 | <0.001 | 117±16.3 | 112±14.5 | <0.001 | 116.5±16 | 116±16 | 0.6 |  |
| eGFR | | 19.2±6.4 | 15±6 | <0.001 | 19.2±6.5 | 15.6±5.8 | <0.001 | 19.24±6.5 | 14.5±5.5 | <0.001 | 19.4±6.6 | 16.6±6 | 0.001 |  |
| BMI | | 29.3±5.9 | 27.5±5.2 | <0.001 | 29.3±5.8 | 28±5.9 | <0.001 | 29±5.8 | 28.5±6 | <0.001 | 29±5.8 | 29.4±6 | 0.105 |  |
| age | | 69±13 | 68.7±13 | <0.001 | 69.3±13 | 68.9±13 | 0.02 | 69.5±13 | 65±13 | <0.001 | 69±13 | 69±12.7 | 0.76 |  |
| S.-Albumin; Serum Albumin(g/l), T.-Calcium; Total Calcium (mmol/l), Phosphate (mmol/l), iPTH (ng/l), Hemoglobin (g/l), eGFR CKD-EPI (ml/min/1.73m^2^) | | | | | | | | | | | | | | |

| **Suppl. Table 2. C-statistic: internal validation** | | |  |  |
| --- | --- | --- | --- | --- |
|  | **C-Statistics** | **95% CIs** |  |  |
| **ESAs** | | |  |  |
| Hb < 100 g/l | 0.572 | 0.465-0.679 |  |  |
| Hb > 100 g/l | 0.720 | 0.670-0.769 |  |  |
| **Iron** |  |  |  |  |
| Hb > 100 g/l | 0.741 | 0.625-0.858 |  |  |
| Hb <100 g/l | 0.607 | 0.547-0.667 |  |  |
| **Phosphate binders** |  |  |  |  |
| Phosphate <1.4 mmol/l | 0.764 | 0.708-0.821 |  |  |
| Phosphate >1.5 mmol/l | 0.598 | 0.485-0.710 |  |  |
| **Phosphate binders** |  |  |  |  |
| iPTH < 150 ng/l | 0.701 | 0.632-0.769 |  |  |
| iPTH > 150 ng/l | 0.741 | 0.635-0.793 |  |  |
| **VDRA** |  |  |  |  |
| iPTH < 150 ng/l | 0.659 | 0.619-0.716 |  |  |
| iPTH > 150 ng/l | 0.668 | 0.590-0.729 |  |  |

| **Suppl. Table 3. Multivariate logistic regression of the risk of requiring any CKD-related pharmacotherapy during the pre-dialysis period** | | |
| --- | --- | --- |
|  | **P value** | **OR^1^ (95% CI)** |
| eGFR at referral | 0.005 | 0.955 (0.93-0.97) |
| Serum albumin > 35 g/l | 0.100 | 1.70 (0.90-3.18) |
| Phosphate >1.5 mmol/l | 0.040 | 1.56 (0.93-2.60) |
| iPTH ref. > 150 ng/l | < 0.001 | 3.04 (1.99-4.05) |
| History of diabetes mellitus | 0.039 | 1.48(1.02-2.15) |

| **Suppl. Table C-statistic: internal validation** | | |
| --- | --- | --- |
|  | **Area** | **95% CIs** |
| Validation cohort | 0.658 | 0.589-0.797 |
| Study cohort | 0.666 | 0.616-0.716 |

| **Suppl. Table 5.** CKD-MBD- and anemia-parameters across the different countries at the end of the follow up. | | | | | | |
| --- | --- | --- | --- | --- | --- | --- |
| Parameter/Country | Bosnia  N=3 | Czech Republic  N= 223 | Italy  N= 68 | Russia  N= 72 | Serbia  N= 46 | Slovak Republic  N= 220 |
| Total calcium (mmol/l) | 2.12$\pm$0.38 | 2.29 $\pm0.25$ | 2.30$\pm0.19$ | 2.33$\pm0.19$ | 2.29$\pm0.18$ | 2.26$\pm0.20$ |
| Phosphate (mmol/l) | 1.37$\pm0.26$ | 1.53$\pm0.4$ | 1.52$\pm0.36$ | 1.46$\pm0.3$ | 1.55$\pm0.44$ | 1.6$\pm0.4$ |
| iPTH (ng/l) | 254$\pm85$ | 168 (198,251) | 167 (177,261) | 161 (184,314) | 158 (141,392) | 129 (147,198) |
| Ferritin (μg/l) | 275$\pm209$ | 346 (452, 601) | 195 (230, 363) | 189 (280, 543) | 422 (405, 836) | 323 (465, 685) |
| Hemoglobin (g/l) | 121$\pm17$ | 112$\pm$17 | 107$\pm13$ | 109$\pm12$ | 104$\pm15$ | 105$\pm19$ |
| *iPTH: intact parathormone. Data are expressed as mean* ± SD or median, interquartile range as appropriate. | | | | | | |
